# Intracellular Biotransformation of Ultrasmall Iron Oxide Nanoparticles and Their Effect in Cultured Human Cells and in Drosophila Larvae In Vivo

**DOI:** 10.3390/ijms23158788

**Published:** 2022-08-08

**Authors:** Alonso Rodríguez Pescador, Lucía Gutiérrez Romero, Elisa Blanco-González, María Montes-Bayón, L. María Sierra

**Affiliations:** 1 Department of Functional Biology (Genetic Area) and Oncology University Institute (IUOPA), University of Oviedo. C/Julián Clavería s/n, 33006 Oviedo, Spain; 2 Institute of Sanitary Research of Asturias (ISPA), Avda, Hospital Universitario s/n, 33011 Oviedo, Spain; 3 Department of Physical and Analytical Chemistry, Faculty of Chemistry, University of Oviedo. C/Julián Clavería 8, 33006 Oviedo, Spain

**Keywords:** ultra-small iron hydroxide adipate/tartrate coated nanoparticles, HPLC-ICP-MS, genotoxicity, cytotoxicity, Caco-2 cells, HepG2 cells, A2780 cells, GM04312 cells, *D. melanogaster*

## Abstract

A systematic investigation on the cellular uptake, intracellular dissolution, and in vitro biological effects of ultra-small (<10 nm) iron hydroxide adipate/tartrate coated nanoparticles (FeAT-NPs) was carried out in intestinal Caco-2, hepatic HepG2 and ovarian A2780 cells, and the nucleotide excision repair (NER) deficient GM04312 fibroblasts. Quantitative evaluation of the nanoparticles uptake, as well as their transformation within the cell cytosol, was performed by inductively coupled plasma mass spectrometry (ICP-MS), alone or in combination with high performance liquid chromatography (HPLC). The obtained results revealed that FeAT-NPs are effectively taken up in a cell type-dependent manner with a minimum dissolution after 3 h. These results correlated with no effects on cell proliferation and minor effects on cell viability and reactive oxygen species (ROS) production for all the cell lines under study. Moreover, the comet assay results revealed significant DNA damage only in GM04312 cells. In vivo genotoxicity was further studied in larvae from *Drosophila melanogaster*, using the eye-SMART test. The obtained results showed that FeAT-NPs were genotoxic only with the two highest tested concentrations (2 and 5 mmol·L^−1^ of Fe) in surface treatments. These data altogether show that these nanoparticles represent a safe alternative for anemia management, with high uptake level and controlled iron release.

## 1. Introduction

The use of iron oxide nanoparticles (IONPs) as potential therapeutic agents in the management of iron-deficiency severe anemia has become an area of increasing importance over the years [1]. Commercially available nano-iron formulations are, in general, spheroidal nanoparticles with an iron oxy-hydroxide core and a carbohydrate shell, differing in size and carbohydrate structure from the core, that can be administered intravenously [2]. They have the ability to quickly fill up the iron deposits in the human body, which is of great importance for patients with critical iron deficiency, i.e., caused by great blood loss, chronic kidney disease or chemotherapy. Trying to overcome the disadvantage associated to the intravenous administration and the adverse event profiles of these formulations, alternative nano-iron agents have been designed and tested [3]. In this regard, some of the most interesting approaches include nanoparticles with iron cores that try to mimic the ferric oxy-hydroxide core of the protein ferritin and coated by biologically compatible ligands [4]. Among them, the ultrasmall (<10 nm) nanoparticles of iron hydroxide adipate tartrate (FeAT-NPs) have been proposed in 2014 as an interesting alternative for oral iron supplementation with a very specific mode of action [5,6]. This nano-compound shows minimum dissolution at the pH of the gastrointestinal tract and reaches the duodenal enterocytes in the almost intact nanoparticulated form. Once there, it is efficiently taken up by the enterocytes as whole nanoparticles by endocytosis, minimizing tissue inflammation, followed by lysosomal dissolution (in a similar fashion to ferritin) to join the intracellular iron pool [7].

Initial experiments to evaluate the extent of intracellular release of iron ions from these ultrasmall FeAT-NPs were conducted in our research group by developing a specific chromatographic separation strategy that permitted to distinguish among the nanoparticulated as well as the iron ionic forms simultaneously [8]. In this work, the aim is to apply the developed strategy to explore the cellular uptake as well as the intracellular dissolution of these FeAT-NPs on a set of different cell models of different origin. Since iron is a potent redox agent, the release of this element from the nanoparticles might involve significant changes on the intracellular redox status. An excess of intracellular soluble iron is toxic, as it generates reactive oxygen species (ROS) by interconverting between ferrous (Fe^2+^) and ferric (Fe^3+^) forms via the Fenton and Haber–Weiss reactions [9]. This adverse effect of soluble iron is a major difficulty in the treatment of anemia. At high levels, ROS may cause oxidative damage to cellular components such as proteins, lipids and DNA [10], which can then trigger cellular cytotoxic and genotoxic responses that are associated with disease [11].

With respect to the induction of DNA damage, previous studies observe genotoxicity with the comet assay, in cultured cells treated with different types of IONPs. For example, silica-coated magnetite (Fe_3_O_4_) nanoparticles were genotoxic in human SHSY5Y neuronal cells [12]; DNA damage was also observed in lymphocytes treated with uncoated Fe_2_O_3_ nanoparticles [13]; and oleic acid-coated magnetite nanoparticles showed a dose-dependent genotoxic activity in astrocytes [14]. However, there are also studies revealing the absence of genotoxicity when using uncoated Fe_2_O_3_ and Fe_3_O_4_ nanoparticles in lung epithelial A549 cells [15], or uncoated magnetite nanoparticles in human lymphocytes and lymphoblastoid TK6 cells [16]. Similarly, thiol-coating Fe_3_O_4_ nanoparticles exposure of human lymphocytes and breast cancer MCF-7 cells caused no increase in DNA damage [17]. Thus, the coating of the particles seems to be a key aspect regarding the associated intracellular solubilization and induced genotoxicity [16].

Therefore, the biological aspects concerning the potential cellular damage caused by the exposure of the different cell lines to FeAT-NPs ought to be, and were, evaluated in this work by addressing cell viability, cell proliferation, ROS formation and induced oxidative DNA damage, and these parameters were correlated with the cellular nanoparticle uptake levels and their intracellular release of iron ions. The use of several human cell lines, with different origins and characteristics, including intestinal, hepatic and ovarian cells (Caco-2, HepG2 and A2780, respectively), and GM04312 cells, known to be deficient in the nucleotide excision repair (NER) system [18], that contributes to the repair of oxidative DNA damage [19,20], would provide a wide background of the evolution of these nanoparticles under different environments, as well as their potential for DNA damage induction [21].

In addition, the effects of these FeAT-NPs were also evaluated *in vivo*, using *Drosophila melanogaster* as a model organism, recommended for testing the genotoxic potential of nanomaterials [22], and the eye-SMART assay to check the induction of somatic mutation and mitotic recombination [23,24,25].

## 2. Results and Discussion

### 2.1. Cellular Uptake and Intracellular Dissolution of Ultra-Small FeAT-NPs

**Figure 1:**
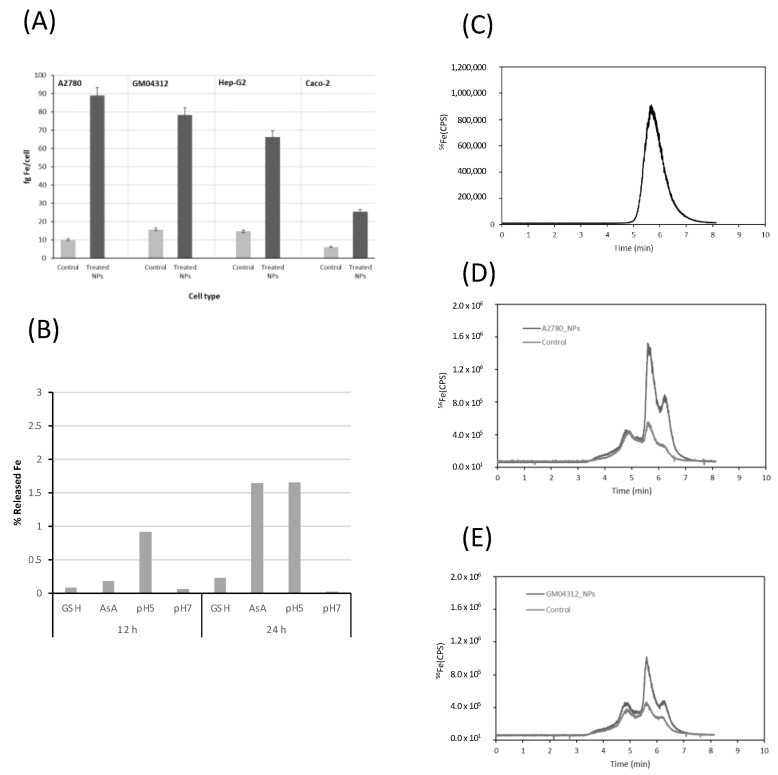
Evaluation of cellular uptake and dissolution of FeAT-NPs by ICP-MS methodologies. (**A**) Uptake of FeAT-NPs in different cell models by monitoring intracellular iron in cell lysates by ICP-MS. Dark grey, cells exposed to 2 mmol·L^−1^ of Fe as FeAT-NPs, for 3 h, and pale grey, non-exposed control cells. (**B**) Iron released from nanoparticles in vitro, incubating them with cell culture medium for 12 and 24 h, under different conditions: with glutathione (GSH), ascorbic acid (AsA), at pH 5 and 7. (**C**) HPLC-ICP-MS chromatogram obtained by monitoring the ^56^Fe signal for synthetic FeAT-NPs. (**D**) HPLC-ICP-MS chromatograms obtained by monitoring the ^56^Fe signal for A2780 cells. Dark grey, cells exposed to 2 mmol·L^−1^ of Fe as FeAT-NPs, for 3 h, and pale grey, non-exposed control cells. (**E**) The same as in (**D**) but for GM04312 cells.

These nanoparticles, described as stable in different conditions and capable of forming small agglomerates, can be taken up by cells [8]. To evaluate their cellular uptake in different cell models, the total Fe concentration was measured by inductively coupled plasma mass spectrometry (ICP-MS) in cell lysates from the different tested cells exposed to FeAT-NPs, at a concentration of 2 mmol·L^−1^ of Fe, for 3 h, and also from the respective non-treated control cells, as described before [8]. The measurement conditions are presented in Table 1. The obtained results are plotted in Figure 1A, expressed as fg Fe per cell. We observed that the intracellular Fe level increased, in all the cell lines, upon exposure to the nanoparticles, which revealed that the FeAT-NPs were effectively taken up by the cells and not merely externally attached to their membranes, as membrane debris from the lysed cells were carefully removed before the measurements. However, the Fe concentration and, thus, the number of nanoparticles taken by cells were closely related to the cell type, as before [8]. As observed, non-treated control cells show similar iron levels, regardless of the cell type (concentrations ranged from 6.5 ± 0.9 fg Fe/cell in Caco-2 cells to 15.5 ± 0.5 fg Fe/cell in GM04312 cells). However, when cells were exposed to FeAT-NPs, the highest increase in iron levels was found in the A2780 cell line, with a final iron concentration of 88.9 ± 6 fg/cell, which is a nine-fold increase over the control. For the other cell lines, the Fe level in treated cells increased around four-fold over the respective controls. Thus, A2780 cells (from ovarian cancer) showed the highest capability to internalize nanoparticles, whereas Caco-2 cells (enterocyte-like cell model) exhibited the significantly lowest FeAT-NPs incorporation.

The differences observed in the uptake of these nanoparticles among cell lines might be attributed to the fact that they enter the cells through endocytosis [26,27]. However, there are different endocytic pathways that are cell-specific and, subsequently, determine the trafficking and intracellular fate of nanoparticles [28,29]. Therefore, the endocytosis efficiency of nanoparticles in mammalian cells is dependent on their physicochemical properties, such as size, shape, and surface chemistry, as well as on the cell type [30,31]. However, in this case, no effect of cell size in the FeAT-NPs uptake was detected, since the largest cells, GM04312, showed a lower uptake level than the smallest ones, A2780.

The next step was to investigate, in vitro first, the release of iron ions from the FeAT-NPs within the cell cytosol. For this aim, nanoparticles were incubated in different media, with two different pH values (physiological, pH = 7, and that characteristic of endosomes, pH = 5). In addition, different cytosolic compounds that might influence Fe release, like glutathione (GSH) and ascorbic acid (ASC), were also tested. After 12 h and 24 h of incubation, the different solutions were ultrafiltered and the mass balance of the retained (thus nanoparticulated) and permeated (thus soluble) fractions was obtained. The observed results are presented in Figure 1B. It can be seen that Fe release occurs more evidently after 24 h and that the presence of ASC and the pH 5 yielded the highest Fe releases, at both 12 and 24 h. In any case, even after 24 h under these conditions, the total Fe released was always below 5% in the individual treatments. It is expected that these values would be slightly higher within the cell cytosol, where most of these conditions occur simultaneously.

To evaluate the possible biotransformation of the FeAT-NPs within the cell cytosol, a modified reversed-phase high performance liquid chromatography (HPLC) separation, using a mobile phase containing sodium dodecyl sulfate (SDS; 10 mM) was used in combination with ICP-MS detection (see Table 1). Previous studies in our research group demonstrated that this speciation/fractionation strategy, initially applied to the separation of Au and Ag nanoparticles [32,33], could be successfully used to separate synthetic FeAT-NPs from aggregates and free iron ions extracted from the cytosol of cells exposed to the nanoparticles [8]. A mixture of gold nanoparticle standards of different sizes (10 and 30 nm), together with ionic gold, were used for the column calibration. Then, the synthesized nanoparticles were injected into the system and the corresponding chromatogram is presented in Figure 1C, where the elution of the nanoparticles can be observed in a single broad peak at about 5.6 min.

This retention time, according to the calibration of the column, might correspond to a size below 10 nm, in the range of 4–6 nm, which is also in agreement with previous results [8].

Figure 1D,E show, as representative examples, the chromatograms obtained for two of the cell lines investigated, the A2780 (Figure 1D) and the GM04312 (Figure 1E), showing results of nanoparticle- treated and untreated cells. Three different fractions can be observed in them at about 4.8, 5.6 and 6.3 min, respectively, which are also present in the control cells (free of nanoparticles). Similar chromatographic profiles were obtained for Caco-2 and HepG2 cells (data not shown). 

The comparison between the chromatograms of Figure 1D,E and that of Figure 1C indicated that the peak at 5.6 min could correspond to the elution of the FeAT-NPs that remained in the native condition (monodispersed) after incorporation into the cell cytosol. Moreover, this peak increased significantly upon exposure of the cells to the FeAT-NPs. In agreement with the total Fe concentration results, this peak was significantly higher in A2780 than in GM04312 cells. On the other hand, the fraction eluting at about 6.3 min, according to the column calibration and previous studies [8], could be associated to the elution of different ionic iron species present in the cell cytosol. This peak also increased upon exposure to the FeAT-NPs and could be ascribed to the release of ionic iron from the nanoparticles within the cell cytosol. The increase, in percentage, of this fraction with respect to the control (the intracellular iron pool contains some of these species even in the control sample) in A2780 cells was of approximately 7.5%. Similarly, in GM04312 cells, the increase in the peak area was about 3.5%. Considering that the chromatograms do not show base-line separation of the different peaks due to the challenging sample, the results revealed a minimum biotransformation of the nanoparticles within the cell cytosol that might be correlated with the previously described low solubilization rate of the particles obtained in in vitro experiments (see Figure 1B). 

Finally, not remarkable changes seem to be observed in the Fe-peak at about 4.8 min that has been ascribed to ferritin (accumulating nanoparticulated iron) but might also be related to nanoparticles-aggregates [8] that seem to be minor in this case.

### 2.2. Biological Effects

#### 2.2.1. Cell Viability and Clonogenic Activity

**Figure 2:**
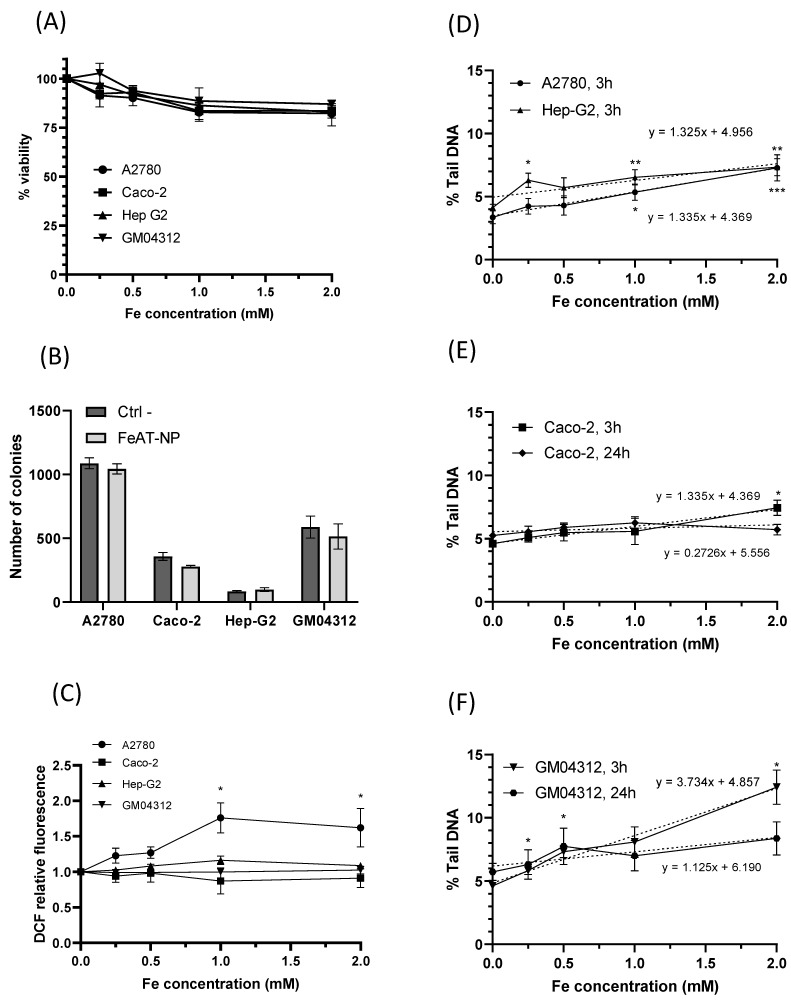
Biological effects of FeAT-NPs *in vitro*. (**A**) Cell viability in Caco-2, HepG2, GM04312 and A2780 cells after 3 h exposure to FeAT-NPs in concentrations ranging from 0 to 2 mmol·L^−1^ of Fe. (**B**) Clonogenic activity results of Caco-2, HepG2, GM04312 and A2780 cells untreated and treated with the nanoparticles at a concentration of 2 mmol·L^−1^ of Fe. (**C**) ROS induction in cells treated for 3 h, at concentrations ranging from 0 to 2 mmol·L^−1^ of Fe: data are expressed as the percentage of DCF fluorescence increase over untreated control cells (as 100%) in Caco-2, HepG2, GM04312 and A2780 cells. TBHP was used as a positive control in these experiments (see text). (**D**) DNA damage, expressed as % Tail DNA, in A2780 and HepG2 cells after 3 h exposure to FeAT-NPs in concentrations ranging from 0 to 2 mmol·L^−1^ of Fe. (**E**) DNA damage, as % Tail DNA, in Caco-2 cells after 3 and 24 h exposure to concentrations from 0 to 2 mmol·L^−1^ of Fe in nanoparticles. (**F**) The same as in (**E**), but for GM04312 cells. * *p* < 0.05; ** *p* < 0.01; *** *p* < 0.001.

To study the biological effects of the FeAT-NPs, cell viability was determined for all analyzed cell lines using the resazurin assay, after 3 h exposure to them at concentrations of 0, 0.5, 1.0, 1.5 and 2 mmol·L^−1^ of Fe. The results, displayed in Figure 2A, show that the increase in nanoparticles dosage resulted in a slight decrease in cell viability, in all cell lines, in a dose-dependent manner, with statistically significant negative regression slopes (b = −7.9, *p* = 0.015 for A2780; b = −7.4, *p* = 0.001 for Caco−2; b = −8.4, *p* < 0.0001 for HepG2; b = −7.5, *p* = 0.001 for GM04312). Cell survival as high as 80% was observed with the highest tested concentration. Therefore, despite differences in FeAT-NPs uptake between individual cell types, no significant differences in viability reduction were detected among cell lines. Moreover, the decreases in viability, although significant, were not biologically relevant for the tested FeAT-NPs concentrations.

The effect of these nanoparticles on cell proliferation was evaluated with the colony formation assay [34], whose results are presented in Figure 2B. Although for all cell lines, except HepG2, the clonogenic efficiency decreased after exposure to FeAT-NPs, none of the differences between treated and untreated cells were statistically significant, for any cell line.

These results revealed that these nanoparticles were not toxic at the tested Fe concentrations, and exerted no significant influence on cell proliferation, in agreement with our previous results in Caco-2 and HT-29 cells [35].

#### 2.2.2. ROS Induction

The ability of FeAT-NPs to induce the production of intracellular ROS in the four investigated cell lines was assessed using, as described in Materials and Methods, a 2,7-dichlorodihydrofluorescein-diacetate (DCFH-DA) fluorescent probe, and *tert*-Butyl hydroperoxide (TBHP) as positive control. As presented in Figure 2C, exposure to these nanoparticles at various Fe concentrations (0.5 to 2 mmol·L^−1^) for 3 h did not induce significant ROS formation in Caco-2, HepG2 and GM04312 cells, when compared to their respective untreated (control) cells, although in Caco-2 cells TBHP, at 200 μmol·L^−1^, induced significant ROS levels (2.259 ± 0.112 fold-times over the negative control). Differences between these data and those found before with these cells [35] may be ascribed to the exposure time (3 versus 48 h). HepG2 and GM04312 cells seemed to be more resistant to ROS formation as slight ROS increases were induced by TBPH (1.258 ± 0.07 fold-times, induced with 400 μmol·L^−1^ in HepG2, and 1.120 ± 0.063 fold-times, induced with 200 μmol·L^−1^ in GM04312 cells). 

In A2780 cells, a statistically significant increase in the level of intracellular ROS was detected with increasing concentrations of FeAT-NPs, up to 1 mmol·L^−1^ of Fe, with a linear regression analysis (y = 0.73x + 0.99, *p* = 0.0002). These results would agree with the higher intracellular iron content detected in A2780 cells compared to the other cell types (see Figure 1A). However, since the induced ROS levels did not double those of the untreated control cells, and they did not increase further at 2 mmol·L^−1^ of Fe, such variation might be punctual. In fact, this ROS increase induced in A2780 cells by the FeAT-NPs was lower than that induced by the positive control TBHP (2.247 ± 0.237), at 50 μmol·L^−1^, which was a rather low concentration as a result of this cell line sensitivity. 

These results demonstrated that the ultrasmall FeAT-NPs, at the studied Fe concentrations, did not relevantly influence the generation of intracellular ROS in the different analyzed human cells, even in sensitive ones like A2780, correlating with the results of cell viability and clonogenic activity.

#### 2.2.3. In Vitro DNA Damage: Comet Assay

To assess the toxicity of a given nanomaterial, the induced DNA damage is an essential parameter that may be measured with the comet assay. This assay, in its alkaline version, detects both single and double strand breaks, alkali-labile sites, stalled replication forks and the activity of DNA excision repair systems [36,37]. Since previous evaluation of the incorporation of the FeAT-NPs into the cell cytosol, by HPLC-ICP-MS, revealed the presence of ionic iron and such a finding was also correlated with a slight increase in ROS in the A2780 cell line, it was necessary to establish if they could induce DNA damage. Preliminary studies using FeAT-NPs in Caco-2 cells concluded that they were not genotoxic [35]. However, in that case, only one concentration (0,25 mmol·L^−1^ of Fe) was tested. 

Therefore, the capacity of ultrasmall FeAT-NPs to induce DNA damage was further assessed by the alkaline comet assay, using the same treatment conditions used for the other studied biological parameters. The obtained results are shown in Figure 2D, for A2780 and HepG2 cells treated for 3 h, Figure 2E, for Caco-2 cells treated for 3 and 24 h, and Figure 2F, for GM04312 cells also treated for 3 and 24 h with the FeAT-NPs. Although the highest tested concentration of nanoparticles (2 mmol·L^−1^ of Fe), after 3 h exposure, induced statistically significant DNA damage in A2780, HepG2 and Caco-2 cells, and the linear dose-response regressions presented significant slopes (*p* = 0.0003 for A2780; *p* = 0.0067 for HepG2; *p* = 0.0031 for Caco-2), the induced damage was rather low, never doubling the spontaneous one. Moreover, in the case of Caco-2 cells, treatments of 24 h did not show any indication of genotoxicity (see Figure 2E). Therefore, treatments with the nanoparticles did not induce relevant DNA damage in these cells. However, when they were treated with the positive control, 250 μmol·L^−1^ methyl methanesulfonate (MMS), high levels of DNA damage (measured as percentages of Tail DNA) were detected in the same experiments (in 3 h exposures: 33.67 ± 4.49 in A2780, 57.16 ± 13.50 in Caco-2, and 70.15 ± 5.63 in Hep-G2 cells; in 24 h exposure: 45.33 ± 6.09 in Caco-2 cells). 

In the case of GM04312 cells (see Figure 2F), these FeAT-NPs induced significant increases in DNA damage (*p* < 0.05) at all tested concentrations, when compared to control cells, with a significant linear dose-response regression slope (R2 = 0.98; b = 3.73 ± 0.49, *p* = 0) in 3 h treatments. In this cell model (see Figure 2F), 24 h exposure did not induce detectable DNA damage at any concentration. Due to this, the regression slope for 24 h exposure was no longer statistically significant (*p* = 0.1256). Higher induced DNA damage was expected in these cells, as compared with the other cell types, and as also detected for the positive control (64.92 ± 1.61% Tail DNA in 3 h exposures, and 66.17 ± 3.21 in 24 h), since GM04312 cells do not repair several types of DNA damage through the NER pathway [18,38], including oxidative DNA damage [19,20,39], when produced by the Fe-generated ROS, even if their levels were low. Furthermore, the decrease on induced DNA damage detected for the highest analyzed concentration, when comparing short and long exposures, might be due to toxicity [40] associated to the lack of DNA repair. 

These results showed that there was no relationship between the intracellular iron concentrations and the DNA damage they induced in different cell types. Altogether these results suggest that not all the FeAT-NPs taken up by cells contributed to increasing the intracellular ionic iron pool, with the subsequent consequences of DNA damage or ROS induction.

#### 2.2.4. In Vivo Somatic Mutation and Recombination: SMART Assay

**Figure 3:**
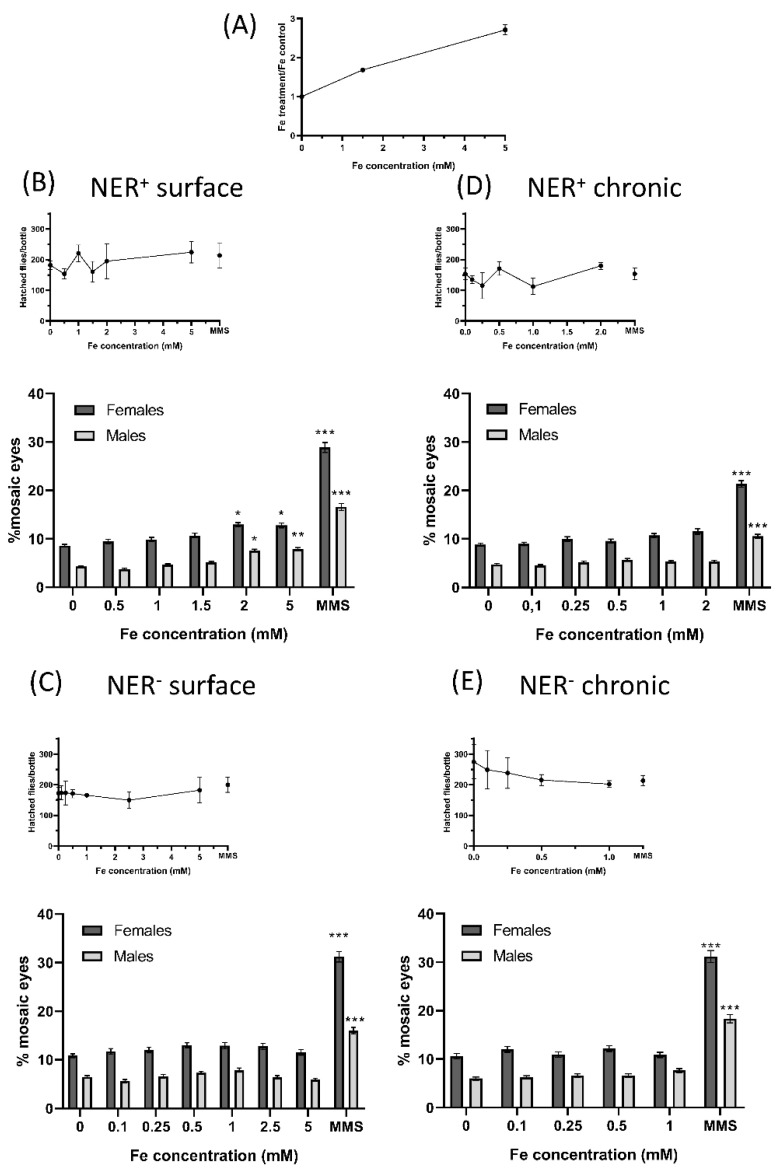
Biological effects of FeAT-NPs *in vivo*. (**A**) Determination of Fe uptake by larvae: Fe content on larvae of *Drosophila* Oregon K strains determined after treating them for 72 h, with 1.5 and 5 mmol·L^−1^ Fe concentrations in FeAT-NPs. (**B**–**E**) Frequencies of mosaic eyes, and of emerged flies (as a semi-quantitative toxicity measurement): (**B**) After surface treatment of 60 ± 12 h old larvae, in NER efficient conditions. (**C**) After surface treatment of 60 ± 12 h old larvae, in NER deficient conditions. (**D**) After chronic treatment in NER efficient conditions. (**E**) After chronic treatment in NER deficient conditions. * *p* < 0.05; ** *p* < 0.01; *** *p* < 0.001.

The results obtained in the in vivo evaluation of FeAT-NPs effects in larvae of *D. melanogaster* are presented in Figure 3 and Table 2 and Table 3.

Regarding the incorporation of the nanoparticles, total Fe determination by ICP-MS revealed an increase of about 1.7-fold in the exposed larvae to 1.5 mmol·L^−1^ Fe, and of about three-fold in the exposed larvae to 5 mmol·L^−1^ Fe, without apparent toxicity (Figure 3A). The number of hatched flies per bottle in both treatments, surface and chronic, in NER efficient and deficient conditions (NER^+^ and NER^−^, respectively), that provided a semi-quantitative estimation of toxicity, showed that these nanoparticles were not toxic, at any of the tested concentrations (Figure 3A–D). However, the chronic treatments of NER^−^ larvae showed a decrease in hatched flies as function of the used concentration (Figure 3D).

In the rest of the analyzed conditions, for some of the nanoparticle treatments, the number of flies that emerged was higher than those of the corresponding negative controls, as if the treatments were a survival stimulus, as described for other types of nanoparticles [25].

The results of the SMART assay are partly presented in Figure 3A–D, as mosaic eyes frequencies, and in Table 2 and Table 3, as the number of spots in 100 eyes, for surface and chronic treatments, respectively.

They demonstrated the lack of risk in the exposure to the FeAT-NPs, since positive genotoxic activity was only detected in NER+ conditions with the two highest tested FeAT-NPs concentrations in surface treatments, and for both analyzed parameters.

Moreover, this activity seemed to be linked to induction of mutations, and perhaps intrachromosomal recombination, since the increases in mosaic eyes detected after these surface treatments were proportionally larger in males than in females. In the case of MMS (positive control), the increases in mosaic eyes were larger in females than males, indicating that MMS induced mutations but also quite high levels of recombination. That only positive results were detected with surface treatments was not unexpected, as higher doses can be used in this mode of treatment, when compared to the chronic one [41]; moreover, in this treatment the exposure times, and also the time for repair of induced damage, are shorter than in the chronic treatments.

When comparing both repair conditions, the frequencies of mosaic eyes were slightly higher in deficient than in efficient conditions for both sexes and even for negative controls. Similar results have been described before [42] and they can be ascribed to the fact that part of the DNA damage, including many of the spontaneous lesions, are repaired by the NER system [43]. However, in this NER− condition, no genotoxic activity was detected after any treatment with the FeAT-NPs.

This lack of NER effect in vivo differs from the results obtained in vitro, with the comet assay. This assay detects DNA damage (strand breaks), whereas the SMART assay detects the consequence of this DNA damage, that is mutations and recombination. Thus, it is feasible that in some cells, the DNA damage is repaired by other systems besides NER (for instance, Base Excision Repair system, BER) before they become the origin of mutations. Furthermore, since the applied doses of nanoparticles were of the same order in both assays, the level of induced DNA damage in vivo should be lower than in vitro and, therefore, easier to repair.

The analysis of the average clone size revealed that the induced damage was fixed as mutations (or recombination) quite late on the larvae development, as the size of most spots was rather small. This fact agrees with an easy repair of induced DNA damage, since only the damage induced immediately before pupation seems to be detected. 

When compared with other iron oxide nanoparticles, or nanomaterials, whose genotoxicity was studied in *Drosophila* [44,45], the FeAT-NPs are the smallest, and the only ones that are non-magnetic, but no relevant differences in genotoxic activity were found among them as larger and magnetic IONPs nanoparticles (<50 nm) showed no dose-dependent genotoxicity, at 1 and 10 mmol·L^−1^ concentrations [44]; whereas, when mixed with nickel, IONPs (30 nm) were genotoxic only at one of the tested concentration (200 μg mL^−1^) [45].

## 3. Materials and Methods

### 3.1. Instrumentation

All ICP-MS (inductively coupled plasma mass spectrometry) experiments during this work were performed using the triple quadrupole instrument iCAP TQ ICP-MS (Thermo Fisher Scientific, Bremen, Germany) working in the single quadrupole (SQ)-hydrogen mode (collision cell mode to eliminate ^40^Ar^16^O and ^40^Ar^16^O^1^H polyatomic interferences) for ^56^Fe^+^ monitoring. The ICP-MS instrument was fitted with a cyclonic spray chamber and a conventional concentric nebulizer.

Chromatographic separations were carried out using the Agilent 1260 HPLC system (Agilent Technologies, Tokyo, Japan) equipped with a Nucleosil C_18_ separation column (7 mm particle size, 250 × 4.6 mm i.d., pore size 1000 Å, Phenomenex, Aschaffenburg, Germany). Detection of iron was performed on-line with the iCAP TQ ICP-MS instrument. The flow from the HPLC was introduced into the ICP-MS instrument via a 15 cm long polyether ketone (PEEK^®^) tube, which was connected to the polytetrafluoroethylene (PTFE) sample tube of the nebulizer

For centrifugation/ultrafiltration steps, a centrifuge Biofuge Stratos Heraeus (Thermo Fisher Scientifc) was used. 

Fluorescence measurements were performed using an Infinite 200 (Tecan, Zürich, Switzerland) microplate reader. Flow cytometry experiments were performed using a CytoFLEX S Flow Cytometer (Beckman Coulter Life Science, Indianapolis, IN, USA).

Nucleoids from the comet assay were photographed, for a posterior quantitative determination of DNA damage, in an Olympus BCX-61 fluorescence microscope, with an Olympus DP70 CCD-coupled camera, from the Scientific and Technical Services (SCTs) of the University of Oviedo.

### 3.2. Chemicals and Materials

All solutions were prepared using 18 MΩ cm deionized water obtained from a PURELAB flex 3 (ELGA VEOLIA, Lane End, UK). Iron (III) chloride hexahydrate (98%, Sigma-Aldrich, Madrid, Spain) was used as the precursor for the nanoparticle synthesis. Sodium tartrate dihydrate (99–101%, Sigma-Aldrich) and adipic acid (99%, Sigma-Aldrich) were solubilized in 0.9% potassium chloride solution (Merck, Darmstadt, Germany) to be used as the nanoparticle coating agents. Ammonium acetate (>98%, Sigma-Aldrich) was used for the synthesis buffer and 5 mol·L^−1^ sodium hydroxide (Merck) was used for the nanoparticle precipitation. Standard solutions of Fe and Ge (1000 mg L^−1^, Merck) were used for total Fe determinations by ICP-MS. Sodium dodecyl sulfate (SDS, 98.5%, Sigma-Aldrich) and ammonium acetate (>98%, Sigma-Aldrich) were used in the mobile phases for the chromatographic separations.

RPMI 1640 Dulbecco’s culture medium, phosphate-buffered saline (PBS) and fetal bovine serum (FBS) were purchased from Gibco (Thermo-Fisher, Spain), and modified Eagle’s medium (DMEM) and trypsin, from Biowest, were supplied by VWR-Avantor (Spain); plasmocin was obtained from InvivoGen (San Diego, EEUU) and methyl methanesulfonate (MMS), tert-butyl hydroperoxide (TBHP), 2,7-dichlorodihydrofluorescein-diacetate (DCFH-DA) were bought from Sigma-Aldrich. Low melting point (LMP) and normal melting point (NMP) agaroses, from Invitrogen, were acquired from Sigma-Aldrich. All other chemicals used were of the highest purity and available from commercial sources.

CellTilter-Blue^®^ Cell Viability Assay Kit was purchased from Promega (Wisconsin, EEUU) and 30,000 Da and 3000 Da Ultra-15 MWCO centrifugal filter units were obtained from Millipore (Darmstadt, Germany).

### 3.3. Synthesis of Ultrasmall Iron Hydroxide Adipate Tartrate Nanoparticles

Ultrasmall iron hydroxide adipate tartrate nanoparticles, FeAT-NPs, (5–10 nm ferric oxy-hydroxide core coated with tartaric/adipic acid) were synthesized according to previous publications [8]. Briefly, an acidic concentrated stock solution of FeCl_3_ was added to a solution containing tartaric acid and adipic acid in 0.9% (*w*/*v*) of KCl to achieve a molar ratio of Fe: adipic acid: tartaric acid in the final suspension of 2:1:1. The initial pH of the mixture was always below 2.0 and the iron was fully soluble. The pH was then slowly increased by dropwise addition of a concentrated solution of NaOH (5 mol·L^−1^) until basic pH for iron precipitation. The entire mixture was then oven-dried at 45 °C for a minimum of 24 h. Purification of the synthetized FeAT-NPs was performed by two centrifugation and ultrafiltration steps using first a 30,000 Da Ultra-15 MWCO centrifugal filter and then a 3000 Da Ultra-15 MWCO centrifugal filter. The size and shape characterization of these nanoparticles was published in previous articles of our research group [8]. To prevent stability problems, fresh particles were synthesized for every experiment of this paper.

### 3.4. Cell Lines, Cell Culture and Drosophila Strains

A2780 (human ovarian carcinoma), Caco-2 (human colorectal adenocarcinoma) and HepG2 (human hepatocarcinoma) cell lines were obtained from the Biotechnological and Biomedical Assays Unit at the SCTs of the University of Oviedo, and GM04312 (immortalized human skin fibroblasts from a XPA gene mutant patient [18] cells were purchased from the NIGMS Human Genetic Cell Repository, Coriell Institute for Medical Research (Camden, NJ, USA).

All these cell lines were cultured in 25-cm^2^ flasks at 37 °C in an atmosphere of 5% CO_2_ and a relative humidity of approximately 95%. A2780 cells were maintained in RPMI 1640 medium supplemented with 10% FBS and 0.2% Plasmocin^®^. Caco-2, HepG2 and GM04312 cells were grown in DMEM medium supplemented with 10% FBS and 0.2% Plasmocin^®^. At 90% confluence, cells were harvested by using trypsin and were subcultured into 25-cm^2^ flasks, six-well plates, or 96-well plates, depending on the experiments to be performed. 

The in vivo analysis of FeAT-NPs effects on larvae of *D. melanogaster* was performed in efficient and NER deficient repair conditions. For the efficient conditions, Oregon-K strains, *yellow* and *white* (*OK-y* and *OK-w*), were used due to their sensitivity to ROS inducing agents [24], and for NER deficient conditions, *mus201* strains, *yellow* and *white* (*mus201-y* and *mus201-w*), were selected as *mus201* is a homologue of the *XPG* gene [46] and, therefore, these flies do not present NER activity.

### 3.5. Quantification of Iron in Cells and D. melanogaster Larvae, and Iron Speciation

Cells were seeded in 25-cm2 flasks, at 1 × 10^6^ cells per flask, and after 48 h, when all of them were at around 80% cell confluence, they were treated with the FeAT-NPs at a concentration of 2 mmol·L^−1^ of Fe for 3 h. After treatment, cells were washed three times with PBS, harvested with trypsin and counted. Cell concentrations varied among cell lines, as a result of their size and growth rate: 7.5–9 × 10^6^ cells for A2780, 9–11 × 10^6^ cells for Caco-2, 5–7 × 10^6^ cells for HepG2 and 3.5–5 × 10^6^ cells for GM04312. Cells were then precipitated by centrifugation to obtain a clean cell pellet. The experiment was made in triplicate for each cell line. Cells not exposed to FeAT-NPs served as negative control in each experiment.

Cell pellets were lysed by addition of 1 mL of cold ultrapure water, followed by five freeze−thaw cycles using liquid nitrogen and a 60 °C water bath. After lysis, cell debris was removed by centrifugation (10,000 *g*, 5 min, 4 °C) and the supernatants were collected and analyzed for total Fe content and Fe speciation. Total Fe concentrations were determined, in aliquots of the cell lysate supernatants previously acidified in 0.1% HNO_3_, by ICP-MS (experimental conditions in Table 1), using a calibration curve obtained by continuous nebulization, after optimizing the sample consumption. Calibration curves were prepared using Ge as internal standard, and diluting Fe and Ge stock solutions (1000 mg L^−1^ in 1% HNO_3_).

For determination of Fe in *D. melanogaster*, second/third instar Oregon-K larvae were treated with 1.5 mmol·L^−1^ and 5 mmol·L^−1^ FeAT-NPs diluted in phosphate buffer (pH = 6.8). Phosphate buffer was used as the negative control. Seventy-two hours after the treatment, using highly concentrated sucrose solutions, larvae were collected in a glass vial, washed with ultrapure water several times, until all of them were clean and at the bottom of the vial, and then they were removed to Eppendorfs. The larvae samples were subjected to an acid digestion with 200 µL of HNO_3_ (65%) for 1 h and 200 µL of H_2_O_2_ (30%) for 4 h until no solid residues were observed. Samples were diluted for further analysis. A Fe calibration curve was performed for the quantification of the iron content in the digested samples.

For Fe speciation studies in the cells cytosols, 100 µL aliquots of the cell lysate supernatants, diluted 1:1 in the mobile phase, were injected into the HPLC-ICP-MS chromatographic system. Separation was carried out in isocratic mode using an ammonium acetate buffer (pH 6.8) solution, containing SDS (10 mmol·L^−1^), as mobile phase at a flow rate of 0.5 mL·min^−1^. Detection was performed by ICP-MS monitoring iron (conditions in Table 1).

### 3.6. Cell Viability Assay

Cell viability was assessed with the resazurin assay, using the CellTilter-Blue^®^ Cell Viability Assay Kit from Promega. The assay is based on the ability of living cells to convert a redox dye (resazurin) into a fluorescent end product (resorufin), with maximum excitation and emission wavelengths of 560 nm and 590 nm, respectively. In brief, cells were seeded in 96-well plates (7500 cells per well for A2780 and Caco-2 cells and 10,000 cells per well for HepG2 and GM04312 cells, as a result of their different proliferation rate) and incubated for 48 h to allow their attachment to the plate. The cells were then treated in triplicate with FeAT-NPs, at different Fe concentrations (from 0 to 2 mmol·L^−1^) for 3 h. A positive control was established using 600 µmol·L^−1^ of MMS. After the treatment, the medium was removed, and the cells were washed with PBS. Then, fresh medium containing 20 µL of reaction mixture from CellTilter-Blue^®^ Cell Viability Assay Kit was added. The plate was shacked for 10 s and incubated using standard cell culture conditions for 4 h, after which fluorescence was measured using a microplate reader (Infinitive 200, Tecan, Switzerland). The percentage of cell viability was calculated from the fluorescence emission values obtained with the microplate reader, using the following equation:

$$\%\;\mathrm{cell}\;\mathrm{viability}=\frac{\mathrm{fluorescence}\;\mathrm{of}\;\mathrm{treated}\;\mathrm{cells}}{\mathrm{fluorescence}\;\mathrm{of}\;\mathrm{control}\;\mathrm{cells}}*100$$

Three independent experiments were performed per cell line.

### 3.7. Clonogenic Activity

The clonogenic activity, defined as the ability of a single cell to grow into a colony (a group of at least 50 cells), was studied with the clonogenic, or colony formation, assay that determines cell reproductive death after treatment [34]. To carry it out, for each investigated cell line, 10^5^ cells per well were seeded in a 6-well plate for 24 h and then treated for 3 h, at 37 °C, in culture medium with FeAT-NPs, at concentrations of 0 and 2 mmol·L^−1^ of Fe. Immediately after treatment, 2000 cells per well were re-plated in new 6-well plates to assess colony forming efficiency. The plates were left in the incubator for 6–10 days, depending on the cell line, until clones of at least 50 cells appeared. The cells were washed with PBS, fixed with methanol: acetic acid (3:1) for 5 min and stained with 0.5% crystal violet in methanol for 15 min. The dye mixture was removed, the plates rinsed with tap water and the colony numbers were counted after drying. Three independent experiments were performed per cell line.

### 3.8. Reactive Oxygen Species Measurement

Intracellular ROS was measured using the cell permeation reagent DCFH-DA, which is a fluorescent dye that can measure hydrogen peroxide, hydroxyl radicals, peroxy radicals and other ROS molecules within the cell. After diffusion into the cell, DCFH-DA is deacetylated by intracellular esterase to generate a non-fluorescent compound, which is rapidly oxidized by ROS to 2,7-dichlorofluorescein (DCF), that is highly fluorescent having a maximum excitation and emission spectra at 495 nm and 529 nm, respectively. The fluorescence intensity is directly proportional to the level of ROS in the cytosol. 

For these experiments, cells were seeded in 6-well plates, at a density of 5 × 10^5^ cells per well, were incubated for 48 h, and then were treated for 3 h with FeAT-NPs, at different Fe concentrations (0–2 mmol·L^−1^). Tert-butyl hydroperoxide (TBHP), at varying concentrations depending on the cells (from 50 to 400 µmol·L^−1^) due to their different sensitivities, was used as positive ROS inducer. After the treatment, cells were harvested with trypsin, centrifuged at 1200 rpm for 10 min, washed with 5 mL PBS and counted. The cells were then incubated with 20 nmol·L^−1^ DCFH-DA, prepared at 2 mmol·L^−1^ in dimethyl sulfoxide (DMSO) and diluted with PBS, at a density of 10^6^ cells per mL, during 30 min in the dark. Cells were then washed thrice, with 10 mL PBS, and were set to a concentration of 10^6^ cells per mL in PBS. The fluorescence was measured by flow cytometry (Cytoflex S, Beckman Coulter) in the FITC channel. Approximately 10^4^ cells per condition were analyzed in each of the three performed independent experiments.

### 3.9. Comet Assay

The alkaline single-cell gel electrophoresis, or comet, assay was performed to determine the DNA damage (i.e., strand breaks, stalled replication forks and alkaline labile sites) as described previously by Collins [36], with slight modifications. After seeding, incubating, and treating the cells as described for ROS measurement, in 6-well plates, cells were collected at a concentration of 1.5 × 10^6^ cells per mL. MMS (250 µmol·L^−1^) was used as positive control. Treated cells were embedded in LMP agarose to a final 0.5% concentration and layered onto slides pre-coated with 0.5% NMP agarose. From this last step on, all the process was performed in darkness or under a red light. After gel solidification, the slides were immersed in cold, fresh lysis solution (2.5 mol·L^−1^ NaCl, 0.25 mol·L^−1^ NaOH, 100 mmol·L^−1^ Na2EDTA, 10 mmol·L^−1^ Tris, pH 10, with 10% DMSO and 1% Triton X-100) for 1 h, at 4 °C. The slides were then placed into a horizontal electrophoresis tank and covered with cold electrophoresis buffer (1 mmol·L^−1^ Na_2_EDTA, 300 mmol·L^−1^ NaOH, pH 13), for 20 min at 4 °C, for DNA unwinding and conversion of alkali-labile sites to single-strand breaks. Electrophoresis was performed in the same buffer at 0.81 V/cm and 300 mA, for 20 min at 4 °C. After electrophoresis, the slides were neutralized three times for 5 min with 0.4 mol·L^−1^ Tris buffer (pH 7.5), fixed in absolute ethanol and air-dried overnight. Slides were then coded for blind analysis and stained with 40 µL of ethidium bromide (0.4 µg mL^−1^) with 1 µL of the fluorescence protector Vectashield^®^ (VECTOR laboratories, Inc. Burlingame). Nucleoids were visualized at 400× magnification with an OlympusBX61 fluorescence microscope, equipped with appropriate filters, and an Olympus DP70 digital camera. Photos taken from 75 nucleoids per slide were analyzed with the interactive automated comet software program KOMET 5 (Kinetic Imaging Limited, now Andor-Oxford Instruments, Belfast, UK). The percentage of DNA in the comet tail (% Tail DNA) was the parameter used to measure DNA damage. For each cell line, two slides were analyzed per FeAT-NPs concentration in each experiment, and three independent experiments were carried out.

### 3.10. In Vivo SMART Assay of Drosophila

This assay monitors in wild-type eyes the presence of white mutant spots, generated by loss of heterozygosity due to point mutations and/or deletions at the white locus, or to mitotic recombination or nondisyunction in heterozygous cells [23,24,47]. Partial discrimination between these endpoints might be achieved comparing data from females and males as spots can be induced in females by all four endpoints (although mostly by mutations and recombination), whereas in males, only mutations and intrachromosomal recombination can induce them [24,41].

To perform the assay, two different treatments were carried out: chronic and surface. For both of them, 50 virgin females *w^+^*/*w^+^* (*yellow* phenotype) were mass mated with 30 males *w*/*Y* (*white* phenotype) and, after 48 h, they were transferred to bottles with instant Carolina Formula 4–24 Drosophila Medium (Carolina, Burlington, NC). In chronic treatments this medium was hydrated with solutions of the different FeAT-NPs concentrations (0–2 mmol·L^−1^ of Fe), in phosphate buffer, pH = 6.8, and the flies were allowed to lay eggs for 24 h. In surface treatments, the Carolina medium was hydrated with phosphate buffer pH = 6.8, the flies were allowed to lay eggs for 24 h and, after 60 ± 12 h, 1.5 mL of the different FeAT-NPs concentrations (0–5 mmol·L^−1^ of Fe), in phosphate buffer pH = 6.8, were added to each bottle. In addition to the negative control (phosphate buffer, pH = 6.8), a positive control, with 2.5 mmol·L^−1^ of MMS, was carried out in each experiment. The eyes of hatched females and males, submerged in solutions with ethanol, tween-80 and water to allow a clear scoring of ommatidia, were observed with a stereomicroscope Leica GZ6 (Leica), at 45x magnification, looking for mutant *white* spots. The eyes with at least one spot (mosaic eyes) were counted, as well as the number of spots per 100 eyes, (counting as independent two spots in one eye if they were separated by four rows of normal ommatidia); in this last case, the spot size, based on the number of affected ommatidia (small: 2 ommatidia, medium: 3–8 ommatidia, large: >8 ommatidia), was also determined. At least 300 eyes per sex and tested condition, from a minimum of two independent experiments, were analyzed. Toxicity was semi-quantitatively estimated counting the number of emerged flies per bottle, in all the tested conditions.

### 3.11. Statistical Analysis

The data are presented as mean values ± standard deviation (SD), or standard error (SE). The differences between non-treated (negative control) and treated cells, in the different experiments and assays, were evaluated with paired and unpaired Student’s t tests. Linearity of dose–response data were checked with linear regression analyses. 

For the *Drosophila* data, the mosaic eyes induced by each nanoparticle concentration, as well as the positive control, were compared to the negative control with Chi square tests. In addition, the Frei–Würgler double-decision chi-square test was applied to the analysis of the number of spots in 100 eyes, with m = 2 for small, medium, and total spots and m = 5 for large spots. In this case, results were expressed as negative (−), positive (+), weakly positive (w+) or inconclusive (i), based on the acceptance, or rejection, of the null (H0) or alternative (HA) hypothesis [42].

## 4. Conclusions

Some pharmacokinetic aspects of nanoparticulated iron products, with regard to their performance in humans, can be modelled by animal and cell-based models, according to the European Medicines Agency. In this work, the incorporation, biotransformation and biological effects of FeAT-NPs have been addressed in cell cultures and in the larvae of *D. melanogaster*. The cellular incorporation has been shown to be cell type-dependent, probably as a function of the endocytic mechanisms, with the highest incorporation in the smallest cells, that is in the A2780 model of ovarian cancer. No precipitation of these nanoparticles was ever detected in any experiment, independently of the exposure time. Metabolic evolution within the cell cytosol revealed minimum solubilization of the incorporated particles after three hours exposure that did not compromise cell viability in any of the studied cell models. In agreement with the elemental speciation studies and to the minimal production of free ionic iron within this period, minimum ROS increase was also observed in all the cells under study. Furthermore, induced DNA damage, detected with the comet assay, was only biologically relevant in the NER deficient fibroblasts model, at the highest exposure concentrations. Thus, although DNA oxidative damage could be occurring, the repairing mechanisms of the cells seem to be efficient to eliminate it in the studied models. Finally, in vivo experiments in larvae of *D. melanogaster* showed some evidence of genotoxicity with the two highest FeAT-NPs concentrations, in surface treatments, in both sexes, but with small increases with respect to the negative control. Overall, the synthetic FeAT-NPs are shown to be safe enough to be used, alone or as carrier, of different drugs in future experiments. Nevertheless, distribution studies in a relevant animal model are essential to evaluate distribution, metabolism and excretion of these nanoparticles and to evaluate the degree of their in vivo degradation or solubilization products.

## Figures and Tables

**Table 1:** Instrumental conditions for Fe measurement in iCAP-TQ-ICP-MS.

Parameter	Value
RF Power [W]	1550
Coolant gas flow [L min^−1^]	14.0
Auxiliary gas flow [L min^−1^]	0.8
Carrier gas flow [L min^−1^]	0.8
Measurement mode	Single Quadrupole
Cell gas flow [mL min^−1^]	0.31(H2)
Q1 bias [V]	0
Qcell bias [V]	−5.94
Q3 bias [V]	−12.0
Q1 masses [u]	Open
Q3 masses [u]	56 (56Fe+)

**Table 2:** Results of the w/w+ SMART assay after surface treatments with different Fe concentrations in FeAT-NPs. Data are presented as the number of spots in 100 eyes. Data of the average clone size and of the number of clones per 10^4^ cells are also presented.

				Number of spots									
Repair		Conc.	Scored	Small		Medium		Large		Total		Spot	Clones/
Status	Sex ^a^	(mM) ^b^	Eyes	N	%	N	%	N	%	N	%	Size ^c^	10^4^ Cells
NER^+^	F	0	734	61	8.31	6	0.82	0	0.00	67	9.13	2.14	4.88
		0.5	392	34	8.67	3	0.77 i	2	0.51 i	39	9.95	2.49	6.19
		1	416	35	8.41	8	1.92 i	0	0.00 i	43	10.34	2.19	5.66
		1.5	412	33	8.01	14	3.40+	1	0.24 i	48	11.65	2.5	7.28
		2	580	70	12.07+	12	2.07 i	2	0.34 i	84	14.48 +	2.34	8.47
		5	510	53	10.39	15	2.94 +	3	0.59 i	71	13.92 +	2.65	9.22
		MMS	402	81	20.15+	46	11.44 +	16	3.98 +	143	35.57 +	4.04	34.67
	M	0	752	28	3.72	5	0.66	0	0.00	33	4.39	2.16	2.37
		0.5	400	14	3.50	1	0.25 i	0	0.00 i	15	3.75	2.06	1.93
		1	412	19	4.61 i	1	0.24 i	0	0.00 i	20	4.85	2.05	2.49
		1.5	430	17	3.95 i	6	1.40 i	0	0.00 i	23	5.35 i	2.26	3.02
		2	542	41	7.56 +	7	1.29 i	0	0.00 i	48	8.86 +	2.06	4.56
		5	508	35	6.89 +	10	1.97 i	0	0.00 i	45	8.86 +	2.36	5.23
		MMS	392	46	11.73 +	19	4.85 +	4	1.02 +	69	17.60 +	3.25	14.30
NER^−^	F	0	610	56	9.18	12	1.97	1	0.16	69	11.31	2.30	3.26
		0.1	316	35	11.08	5	1.58 i	3	0.95 i	43	13.61	3.30	5.62
		0.25	308	31	10.06	6	1.95 i	2	0.65 i	39	12.66	3.21	5.07
		0.5	492	61	12.40	10	2.03 i	1	0.20 i	72	14.63	2.27	4.09
		1	310	33	10.65	10	3.23 i	0	0.00 i	43	13.87	2.28	3.95
		2.5	298	30	10.07	8	2.68 i	3	1.01 i	41	13.76	4.05	6.96
		5	304	27	8.88	9	2.96 i	0	0.00 i	36	11.84	2.33	3.45
		MMS	410	102	24.88	62	15.12 +	45	10.98 +	209	50.98 +	5.25	33.48
	M	0	510	32	6.27	5	0.98	1	0.20	38	7.45	2.38	2.28
		0.1	302	15	4.97	1	0.33 i	1	0.33 i	17	5.63	2.41	1.70
		0.25	302	19	6.29	2	0.66 i	0	0.00 i	21	6.95	2.19	1.90
		0.5	494	33	6.68	6	1.21 i	0	0.00 i	39	7.89	2.13	2.10
		1	280	16	5.71	5	1.79 i	1	0.36 i	22	7.86	3.05	2.99
		2.5	282	17	6.03	0	0.00 i	1	0.35 i	18	6.38	2.89	2.31
		5	306	18	5.88	2	0.65 i	0	0.00 i	20	6.54	2.10	1.72
		MMS	406	56	13.79 +	29	7.14 +	7	1.72 +	92	22.66 +	3.19	9.05

^a^: F, females; M, males. ^b^: MMS at a concentration of 2.5 mM, ^c^: Average spot size. Small spots: 2 ommatidia; medium spots: 3–7 ommatidia; large spots: ≥8 ommatidia. N, number of spots. +, positive genotoxic activity; i, inconclusive genotoxic activity.

**Table 3:** Results of the w/w+ SMART assay after chronic treatments with different Fe concentrations in FeAT-NPs. Data are presented as the number of spots in 100 eyes. Data of the average clone size and of the number of clones per 10^4^ cells are also presented.

				Number of spots									
Repair		Conc.	Scored	Small		Medium		Large		Total		Spot	Clones/
Status	Sex ^a^	(mM) ^b^	Eyes	N	%	N	%	N	%	N	%	Size ^c^	10^4^ Cells
NER^+^	F	0	682	67	8.57	9	1.15	0	0.00	76	9.72	2.15	5.28
		0.1	512	39	7.62	9	1.76 i	1	0.20 i	49	9.57	2.45	5.86
		0.25	350	27	7.71	6	1.71 i	1	0.29 i	34	9.71	2.97	7.85
		0.5	428	33	7.71	11	2.57 i	1	0.23 i	45	10.51	2.76	7.25
		1	542	52	9.59	8	1.48 i	2	0.37 i	62	11.44	2.38	6.81
		2	208	33	10.71	4	1.30 i	0	0.00 i	37	12.01	2.32	6.97
		MMS	534	103	16.25 +	42	6.62 +	17	2.68 +	162	25.55 +	3.97	24.53
	M	0	592	31	4.48	2	0.29	0	0.00	33	4.77	2.11	2.49
		0.1	442	18	4.07	2	0.45 i	0	0.00 i	20	4.52	2.19	2.35
		0.25	310	16	5.16 i	1	0.32 i	0	0.00 i	17	5.48 i	2.06	2.99
		0.5	352	15	4.26	5	1.42 i	0	0.00 i	20	5.68 i	2.45	3.48
		1	508	27	5.31 i	1	0.20 i	0	0.00 i	28	5.51	2.04	2.81
		2	208	14	4.52	2	0.65 i	0	0.00 i	16	5.16	2.09	2.76
		MMS	502	49	8.14 +	18	2.99 +	2	0.33 i	69	11.46 +	2.53	6.80
NER^−^	F	0	302	30	9.93	6	1.99	0	0.00	36	11.92	2.19	3.27
		0.1	308	30	9.74	5	1.62 i	3	0.97 i	38	12.34	3.21	4.95
		0.25	302	30	9.93	4	1.32 i	1	0.33 i	35	11.59	2.40	3.48
		0.5	304	31	10.20	6	1.97 i	2	0.66 i	39	12.83	3.97	6.37
		1	304	32	10.53	3	0.99 i	0	0.00 i	35	11.51	2.11	3.04
		MMS	308	68	22.08 +	37	12.01 +	12	3.90 +	117	37.99 +	4.55	21.61
		0	300	18	6.00	2	0.67	0	0.00	20	6.67	2.10	1.75
		0.1	306	18	5.88	1	0.33 i	0	0.00 i	19	6.21	2.06	1.59
	M	0.25	304	22	7.24 i	1	0.33 i	0	0.00 i	23	7.57 i	2.04	1.93
		0.5	304	19	6.25 i	4	1.32 i	0	0.00 i	23	7.57 i	2.19	2.07
		1	302	23	7.62 i	2	0.66 i	0	0.00 i	25	8.28 i	2.12	2.19
		MMS	300	43	14.33 +	15	5.00 +	3	1.00 i	61	20.33 +	3.08	7.84

^a^: F, females; M, males. ^b^: MMS at a concentration of 0.25 mM, ^c^: Average spot size. Small spots: 2 ommatidia; medium spots: 3–7 ommatidia; large spots: ≥8 ommatidia. N, number of spots. +, positive genotoxic activity; i, inconclusive genotoxic activity.

## Data Availability

Not applicable.
